# Supplementary material for: In Silico and In Vitro Analysis of Sulforaphane Anti-Candida Activity
Source: Antibiotics (Basel). 2022 Dec 19;11(12):1842. doi: 10.3390/antibiotics11121842 (PMC9774275; doi:10.3390/antibiotics11121842)
Supplement: Supplementary file 1 [file antibiotics-11-01842-s001.zip › antibiotics-2036393-supplementary.pdf]

Supplementary Table S1. Minimum Inhibitory Concentration (MIC; µg/ml) and Minimum Fungicidal Concentration (MFC; µg/ml) values of sulforaphane (SFN) compared to fluconazole (FLZ) against non-*Candida albicans* strains and clinical isolates.

Strain	MIC (µg/mL)		MFC (µg/mL)	
	SFN	FLZ	SFN	FLZ
C. parapsilosis ATCC 22019	30	4	30	32
Oral 01 HIV+ *C. parapsilosis*	60	4	60	32
C. krusei ATCC 6258	7.5	16	7.5	16
Oral 22 *C. krusei*	30	16	30	> 64
C. glabrata ATCC 2001	30	4	30	> 64
Vaginal 11 *C. glabrata*	30	16	30	64
Vaginal 14 *C. glabrata*	1.87	16	1.87	32

Supplementary Table S2. *In silico* identification of additional non-antimicrobial activities of sulforaphane (SFN) and fluconazole (FLZ).

SFN			FLZ		
**Non-antimicrobial activities**	**Pa**	**Pi**	**Non-antimicrobial activities**	**Pa**	**Pi**
CYP2E1 inhibitor	0.986	0.001	Cytochrome P450 inhibitor	0.740	0.004
Glutathione S-transferase substrate	0.961	0.002			
Apoptosis inducer	0.950	0.003			
Chemoprotective	0.915	0.001			
Cytoprotectant	0.887	0.001			
NF-E2-related factor 2 stimulant	0.882	0.000			
HMOX1 expression enhancer	0.833	0.003			

Pa: probability of a compound being active; Pi: probability of a compound being inactive.
